# Hospitals and Milkmen

**Published:** 1897-02-06

**Authors:** 


### HOSPITALS AND MILKMEN.

Writing in reference to the milk supplied to hospitals, a correspondent says: It is well known that contracts with large institutions are taken at very low prices by the wholesale dealers, and yet are eagerly sought and tendered for. Why? Simply because in many instances they form convenient outlets for a large overplus of milk. An ignorant person will often be heard to say, "What beautiful milk this is." They judge simply by its appearance. To the eye it has a rich colour, and apparently is, as the public would say, "full of cream." But what is the explanation. Simply that it is artificially coloured with annatto. In this magical word lies the principal danger to the hospital. The dealer with a full knowledge of its power, is able to "fake up" any poor milk, and so fulfil his contract. Stale, or slightly sour, milk is "doctored" by the use of borax. Here it is, then, that the big contract taken at a low price comes in, and the institution is made the outlet for the wholesale dealers' "returns"; that is, the milk brought back by the carmen when they have completed their daily round. What the authorities should do, therefore, is to have an analysis of the article supplied frequently taken, and so do away with the possibility of their being supplied with anything but the genuine article. Let us next take the question of quantity. Milk at large institutions is received in gauged drums, or what are known as "milk receivers," the receivers having down their sides a gauge piece, a reference to which will show at once the quantity that there is in it at any time. These "receivers" are, however, readily converted into "deceivers" by a very simple process. All that the carman delivering the milk has to do is to take the "receiver" round the corner, where his process of converting is not seen, and force up the bottom with a friendly kick, or by the same means put a good large dent in the side. The older the drums and the more dents in it the better for the seller and worse for the buyer, for every dent takes away from the holding capacity, and thus it can be seen that there is here a grand opportunity for the exercise of that art of giving what is called "short measure." Let every hospital or charitable institution purchase a Government stamped measure and, after the milkman has called, measure with it the quantity of milk he is supposed to have delivered into the "receiver," not once but frequently, and we think it may possibly learn something of advantage. This should be done personally by the secretary as an additional check. I do not mean that the secretary need actually himself wield the measure, but it should be done under his eye. In conclusion, I commend these hints to every board of management. I have taken milk merely as an illustration; there is a large field for inquiry in other articles, for, sad though it may be, true it is that charitable institutions run more risk of loss from this kind of thing than any other.

*** There can be no doubt of the necessity of keeping a sharp look out in regard to the points referred to by our correspondent. In the report of the Local Government Board for 1893-94 special attention is drawn to the matter; several instances are given of the gross adulteration to which milk supplied to hospitals, orphanages, and asylums is subject, and it is said, "These instances prove the necessity for a strict watch being kept by the governing bodies of such institutions in order to ensure the purity of the milk supplied to the inmates under their charge." The bulging of measure is certainly an ingenious way of perpetrating short measure.—Ed. *T. H.*

M. D. M. writes to ask how a newly-qualified man (M.B., T.C.D.) should set about trying to secure a position as house surgeon in one of the London hospitals, and also asks whether any salary is generally attached to the office.

*** It is out of the question for an outsider to think of becoming house surgeon at any of the hospitals in London which are connected with medical schools, the position being one of the highest prizes for the students connected therewith. There are, however, many hospitals which are not connected with any medical school, and at most of these a salary is given. Vacancies and the persons to whom application is to be made are announced by advertisement.—Ed. *T. H.*

